# The New Normalcy in Dentistry after the COVID-19 Pandemic: An Italian Cross-Sectional Survey

**DOI:** 10.3390/dj9080086

**Published:** 2021-07-31

**Authors:** Stefano Salgarello, Matteo Salvadori, Francesco Mazzoleni, Jacopo Francinelli, Paolo Bertoletti, Elisabetta Audino, Maria Luisa Garo

**Affiliations:** Department of Medical and Surgery Specialties, Radiological Sciences and Public Health, Dental School, University of Brescia, 25123 Brescia, Italy; stefano.salgarello@unibs.it (S.S.); salvaz.dori@gmail.com (M.S.); framaximo@tiscali.it (F.M.); jacopo.francinelli@gmail.com (J.F.); paolobertoletti1993@gmail.com (P.B.); bettaz92@gmail.com (E.A.)

**Keywords:** COVID-19, new normalcy, survey, dentists’ perception

## Abstract

Background: After the first lockdown, Italian dentists resumed their practice while handling several challenges. Reducing contagion risk by complying with the stringent measures recommended by the Italian Ministry of Health for dental activity while also balancing patient needs was a difficult task. This work aims to understand the procedures that were adopted in the second phase of the COVID-19 pandemic (5 May–30 September 2020) and the dentists’ expectations and concerns about returning to normalcy. Methods: A national survey with 38 questions was conducted from November 2020 to January 2021 and comparisons were performed among the five main Italian geographic areas. Results: Located mainly in northwest Italy, 1028 dentists were included in the survey. About 83% of the Italian dentists fully restarted their activities after the lockdown. The resumption was significantly marked in North Italy and the Center than in the South (*p* < 0.01). Over 80% adopted the recommended precautional guidelines, modifying them according to the specific dental treatment executed. Fifty percent of dentists were confident in returning to normalcy after the COVID-19 crisis. Many precautions adopted during the pandemic will be continued, especially in South Italy and the Islands (*p* < 0.01). Conclusions: Italian dentists reported excellent autonomous organizational skills and the maintaining of high-quality precautions during the reopening phase.

## 1. Introduction

According to the World Health Organization (WHO), over three million confirmed cases and 100,000 deaths had been reported in Italy from the beginning of the COVID-19 pandemic [1]. Radical changes have occurred in dentistry during the last year; the way in which dental services are provided has been modified, and new challenges await dentists worldwide.

The practice of dentistry has always exposed dental health professionals to infectious disease agents [2] due to the proximity to the patient’s mouth and the use of aerosol-generating procedures [3]. The SARS-CoV-2 outbreak has further increased the risk of infection. In 2020, the Occupational Safety and Health Administration (OSHA) included dental health professionals in the “very high exposure risk” category [4]. The production of droplets and aerosols during dental treatments, the propinquity and direct contact with potentially infected mucosa, and the use of procedures that may induce gagging or coughing of patients can facilitate the transmission of COVID-19 [5]. 

From the beginning of the pandemic, the Centers for Disease Control and Prevention (CDC) and the American Dental Association (ADA) provided several protocols and guidelines to reduce contagion risk during dental treatments [6,7]. The key elements of these protocols are reducing the risk using physical barriers between the patient and provider, increasing the use of personal protection equipment (PPE), and adopting more effective instrument sterilization methods and environmental reprocessing, especially when positive or suspected patients are treated. 

Telephone triage, the COVID-19 questionnaire about patients’ medical history during the last 14 days before dental treatment, and body temperature measurements have been recommended to reduce and contain the risk of COVID-19 infection [8,9]. Nevertheless, these procedures may not be sufficient: asymptomatic or pre-symptomatic patients cannot be detected [10,11] and antigen COVID-19 tests, commonly used to rapidly identify infected patients, can produce false-negative results [12]. 

In March 2020, given the rise of infected cases, the Italian government imposed a prolonged lockdown [13]. High levels of assistance were ensured for both urgent and non-urgent treatments. Urgent dental treatments were handled, taking all recommended precautions to prevent COVID-19 infection, while non-urgent dental treatments were managed by telephone and, when possible, deferred. Nevertheless, an increasing sense of uncertainty and concern about the effective restarting of dental activity remained after lockdown [14]. A recent survey demonstrated that 57.2% of the dentists were not trained sufficiently to restart after lockdown [15]. Although most of them were trained in infection prevention procedures (64.3%), their capacity to prevent the spread of COVID-19 was strongly reduced [15]. 

After the end of the lockdown in May 2020, dental offices gradually restarted their everyday activities without restrictions, although new COVID-19 waves had been continuing to impact the Italian population. On 30 May 2020, the Italian Ministry of Health released the “Operational guidelines for dental activity during Phase 2 of the COVID-19 pandemic” to provide procedural clinical indications to minimize the risk of transmission in the dental offices [16]. Since the end of 2020, multiple variants of the SARS-CoV-2 virus have been circulating in Italy, thus, increasing the pressure on dental activity once again. In Italy, the prevalence of the so-called “English variant” (technically called B.1.1.7) is currently 17.8% (range: 0–59%) with a level of contagiousness between 30–50% [17]. 

This study aimed to describe the normalcy of private dental offices after the first Italian lockdown (5 May–30 September 2020) and the new normalcy of dentistry after one year of the COVID-19 outbreak. A national survey was conducted from November 2020 to January 2021. Comparisons among different Italian geographical areas were determined to consider the context within which dental teams were operating.

## 2. Materials and Methods

A cross-sectional study was designed on the management of dental offices after activity resumption from May 2020. A structured online questionnaire was conducted from November 2020 to January 2021 through a Google Form. 

The questionnaire was addressed to Italian dentists and distributed through the authors’ mailing lists and Italian dental associations. Participation in the questionnaire was voluntary, anonymous, and without any form of remuneration. This study did not fall under human research’s Italian law and the Ethical Committee did not ask for specific approval. The Ethics Committee of Brescia granted an exemption for this study reporting the following reason: “study is an observational study where all the data have been collected in an anonymous manner.”

In the invitation email, we explained our research purposes and that the University of Brescia was responsible for data collection and management. We specified that the project and its findings were to be published in scientific articles.

Survey questions were developed after reviewing the pertinent literature [18,19]. The questionnaire was designed in the Italian language and comprised 38 single or multi-choice questions (see Supplementary Materials S1). The validity of the questionnaire was determined by consulting five experts (dentists and professors at the Dental School of the University of Brescia) and through a pre-test conducted on 20 dentists chosen randomly in the interest population. Survey length, question suitability, and non-ambiguity of the definitions were considered. The sample size was determined a priori (population: 57,000 dentists, confidence level: 95%, margin of error: 5%; sample size required: 387 respondents). A reminder mail was sent three times after the first invitation.

The data was exported in an Excel file (Microsoft Corp., Redmond, WA, USA) and analyzed with STATA16 (Stata Corp., College Station, TX, USA). According to the ISTAT (National Institute of Statistics) definition [20], geographic areas were divided into five official regions: North-East, North-West, Center, South, and Islands (details about the number of dentists in each area is reported in Supplementary Materials S2). We performed a comparison on all variables among the aforementioned mentioned five geographic areas. For the perception, we also implemented a comparison related to age range. Descriptive statistics were reported as mean ± standard deviation for quantitative data and as frequencies and percentages for qualitative data. The Kruskal–Wallis test with Dunn’s procedure and the chi-square test were performed. Statistical significance was set at 5% (*p* < 0.05). 

## 3. Results

One thousand and twenty-eight dentists (1028) were included in the survey (71.75%, 734/1023, males and 28.25%, 289/1023, females) (response rate: 41.94%; the percentage of missing answers ranged from 0.09% to 0.48%).

Thirty-eight (389/1027) percent of the respondents were aged between 46 and 60 years, 28.24% (290/1027) over 60 years, 26.68% (274/1027) aged between 30 and 45 years, and only 7.21% (74/1027) were less than 30 years. About 70% (711/1028) worked in northwest Italy, while 11.77% (121/1028), 9.24% (95/1028), 6.81% (70/1028), and 3.02% (31/1028) worked in the Center, South, North-East, and the Islands, respectively (Table 1).

### 3.1. Management of the Dental Activity after the Lockdown

More than 50% (518/1028) of the respondents treated less than ten patients daily after the resumption of their activity while about 42% (436/1028) treated between 11 and 20 patients per day. From the end of the lockdown, 82.86% (850/1028) of dentists restarted their working activity. This resumption was marked in many Italian regions, except in the South, where about two-thirds (69.47%, 66/95) of the dentists completely restarted their dental activity (c^2^ (4) = 14.29, *p* < 0.01). Complete results are reported in Table 2.

Seventy-eight percent (801/1028) of the dentists changed the FFP2 mask after every eight hours (Table 3A) and 61.58% (633/1028) simultaneously wore FFP2 and surgical masks, especially in the North (North-West: 62.87%, 447/71; North-East: 70%, 49/70), and in the Center (61.98%, 75/121) (*p* < 0.05) (Table 3B). Only 22.08% (227/1028) of dentists replaced the FFP2 mask after each aerosol-generating procedure, mainly in the South (38.95%, 37/95) and the Islands (48.39%, 15/31) than in the other areas (Center: 21.49%, 26/121; North-East: 20.00%, 14/70; North-West: 18.99%, 135/711) (c^2^ (4) = 32.33, *p* < 0.001) (Table 3C).

For aerosol-generating treatments, 85.21% (876/1028) of the respondents wore a cap, 81.42% (837/1028) a disposable over coat, 92.02% (946/1028) wore face shields or protective eyewear. Shoe covers were used mainly in the South (30.53%, 29/95) and the Islands (22.58%, 7/31) than in other regions (North-West: 14.06%, 100/711; North-East: 12.86%, 9/70; Center: 21.49%, 26/121) (c^2^ (4) = 20.17, *p* < 0.001) (Table 3D).

Eighty-nine (915/1028) percent of the dentists ventilated the treatment room after each patient (Table 3E) and about 45% (461/1028) executed air sanitization with specific devices after each aerosol-generating procedure (Table 3F). 

Telephone triage, patients’ hand disinfection, body temperature measurement, and completion of the COVID-19 questionnaire were practiced by 89.40% (919/1028), 99.51% (1023/10289), 97.37% (1001/1028), and 92.02% (946/1028) of the dentists. 

About 76% (778/1028) executed further disinfection of patients’ hands before and after accessing the dental treatment room.

The average time of waiting before accessing the dental treatment room ranged between five and 10 min (46.50%, 478/1028) and less than five minutes (40.76%, 419/1028). About 11% (114/1028) and 1.65% (17/1028) of the dentists reported an average time of waiting higher than 10 or 15 min, respectively, with differences among the geographical locations (c^2^ (4) = 28.25, *p* < 0.01). 

About 89% (914/1028) executed preliminary mouth rinses. Hydrogen peroxide and chlorhexidine, used in sequence, were reported as the preferred mouthwash by over 53% (490/914) of the respondents. This approach was mainly adopted in the North-West (57.48%, 365/637) and the South (51.16%, 44/86) than in the North-East (38.33%, 23/60), where dentists preferred mouth rinses with chlorhexidine (30.00%, 18/60) or hydrogen peroxide alone (20.00%, 12/60). 

Complete results about patients’ management are describe in Table 4.

### 3.2. Contagions and Management of Positive Patients 

The SARS-CoV-2 virus infected about 9% (91/1028) of the respondents: the prevalence was higher in the North-West (10.55%, 75/711), North-East (10.00%, 7/70), and the Islands (9.88%, 3/31) than in the Center (3.31%, 4/121) and the South (2.11%, 2/95) (*χ*^2^ (4) = 12.65, *p* < 0.05). In the North-West and North-East, higher percentages of contagion were reported among interviewees’ household members (North-West: 15.75%, 112/711; North-East: 17.14%, 12/70, *χ*^2^ (4) = 25.29, *p* < 0.001) and staff members (North-West: 23.91%, 170/711; North-East: 17.14%, 12/70; c^2^ (4) = 25.78, *p* < 0.001) (Table 5A). About 44% (375/860) of the dental owners asked for serological tests for their employees at the end of the lockdown (Table 5B). 

Only 21.60% (222/1028) of dentists treated positive or suspected patients after the resumption of dental activity. A higher percentage of positive or suspected patients were treated in the North-West (22.78%, 162/711), North-East (24.29%, 17/70), and Center (23.14%, 28/121). About 28.85% (64/222) of the respondents adopted additional precautions than those prescribed by current guidelines to treat positive patients (Table 5C), such as wearing the FFP3 mask or treating positive patients at the end of the working day.

Fourteen percent (143/1028) of the dentists treated patients who were declared positive after one or two days after dental treatment (Table 5D). In these cases, more than half (74/143) of the total number required advice from their competent doctor to decide possible additional precautions, while the remaining 48% (69/143) decided independently. In both cases, over 60% chose not to adopt any extra protection considering the adopted procedures suitable to prevent contagion. Among those who contacted a competent doctor, 20.27% (15/74) decided on the rapid antigen test and quarantine for the entire dental team who came into contact with the positive patient. Among those who made autonomous decisions, 23.19% (16/64) of dentists opted to execute the rapid antigen test only (Table 5E).

### 3.3. Concerns

From the analysis of the dentists’ concerns after the first lockdown (Table 6), a moderate level of anxiety emerged about the risk of contagion during the working activity (3.06 ± 1.33) with a statistically significant difference among the geographical areas (North-West: 2.98 ± 1.32; North-East: 2.83 ± 1.43; Center: 3.17 ± 1.25; South: 3.55 ± 1.40; Islands: 3.45 ± 1.18; *χ*^2^ (4) = 20.80; *p* < 0.001) and age range. A high level of anxiety was reported about the need of quarantine in case of COVID-19 contagion or contact with a positive patient (3.74 ± 1.36). Financial concerns were more perceived in the South (4.17 ± 1.08) and Islands (3.96 ± 1.11) than in the North-West (3.44 ± 1.32), North-East (3.31 ± 1.37), and Center (3.66 ± 1.34) (*χ*^2^ (4) = 33.69, *p* < 0.001). Suspending dental activity because of an increase in the rate of contagion was not considered a source of anxiety (1.58 ± 1.07). A high level of trust was reported regarding the current procedures to prevent contagion (3.90 ± 1.10). 

Older dentists reported more concerns about the possibility to contract the virus (3.26 ± 1.33, *χ*^2^ (3) = 11.94, *p* < 0.01) and to continue to have economic losses in 2021 (3.94 ± 1.20, *χ*^2^ (3) = 74.68, *p* < 0.001) and indicated the suspension of their activity as a possible solution to handle the current pandemic situation (1.74 ± 1.17, *χ*^2^ (3) = 12.24, *p* < 0.01). Dentists in the age range of 46–60 years reported to be confident in contagion prevention procedures (4.05 ± 1.03, *χ*^2^ (3) = 13.41, *p* < 0.01). Complete results are reported in the following Table 7.

### 3.4. Return to Normalcy: Procedures and Expectations

About 50% (505/1028) of the interviewees believed that dental activity could return to pre-COVID normalcy (Table 8A). The current precautional behaviors will be maintained by over 90% (459/505) of dentists even when the pandemic will be under control (Table 8B). Room ventilation (37.94%, 390/1028), incorporating face shields (36.67%, 377/1028), and patients’ hand disinfection (33.95%, 349/1028) were indicated as useful future precautions. The FFP2 mask was considered an essential PPE in aerosol-generating treatments (32.78%, 337/1028), and its use will continue in the future. After the COVID-19 pandemic, more extensive use of the FFP2 mask was expected in the Center (43.80%, 53/121), South (40.00%, 38/95), and Islands (54.85%, 17/31) (*χ*^2^ (4) = 20.16, *p* < 0.001) (Table 8C). 

The new normalcy concept was intended to provide greater relaxation during the entire daily activity (32.49%, 334/1028) and patient flow restoration as in the pre-COVID period (28.60%, 294/1028). The reduction of anxiety or fear of contagion was indicated as a critical element for returning to normalcy by 25% (257/1028) of the dentists, especially in the Islands (38.71%, 12/31), South (35.76%, 34/95), and Center (33.88%, 41/121) (*χ* ^2^ (4) = 18.71, *p* < 0.01) (Table 8D).

## 4. Discussion

Since the beginning of the COVID-19 pandemic, Italian dentists have been exposed to a high level of anxiety and stress because of the rapid spread of the SARS-CoV-2 infection in the Italian peninsula and the need for quick adaptation to the new COVID-19 guidance for dental settings [21,22]. 

This study aimed to describe the procedures adopted after the first Italian lockdown (3 March–4 May 2020) and the dentists’ expectations and concerns after one year of the COVID-19 outbreak. 

Approximately 80% of Italian dentists resumed their regular dental activity after the first lockdown, although with some geographical differences due to the different evolution of the virus over time (*p* < 0.01). The reopening rate worldwide varies from 36% in the UK to 47% in Palestine [23]. Our findings described a different and optimistic scenario in the Italian peninsula in line with that described in the USA, where 99% of dental practices are reopened [24].

From our analysis about a possible geographical difference in reopening rate, we observed that the percentage of reopening remained high in the areas such as the South of Italy and the Islands, where the spread of the virus was restrained during the first wave and dramatically increased during the following reopening phase [25]. However, this slight decrease reflects the timeline in regional outbreaks and the speed with which local health systems responded to COVID-19, a phenomenon that already emerged globally in Bakaeen et al. (2021) [18]. 

Overall, Italian dental offices emerged as safe places where high levels of precaution in implementing the security standards played a crucial role in reducing contagion risk. Approximately 80% of dentists adopted all recommended precautional guidelines, modifying them according to the specific dental treatments. Furthermore, over 90% of the respondents applied CDC recommended guidelines before patients’ admission, resulting consistently with evidence reported by Estrich et al. (2021) in the USA [26]. Our findings have confirmed the good level of scientific knowledge of Italian dentists about the characteristics of the coronavirus and the precautionary measures needed to limit the spread of the virus in dental environments [27]. 

The high standards of the restricted protective measures adopted in the Italian dental offices were confirmed in managing positive patients. In these cases, to improve safety for the whole dental team, about 30% of dentists wore additional PPE, used more sanitation and ventilation procedures than those recommended by the guidelines and ministerial dispositions [16], preferred to treat positive or suspected patients at the end of the working day, and wore an FFP3 mask during the treatment. Although a small percentage of our sample had treated positive patients or those who tested positive after the dental treatment, a low level of infections among respondents emerged in line with the rate of the COVID-19 incidence globally reported among dentists, which ranges between 0.9% and 1.1% [26,28,29], except in certain situations (5.3%), as observed in Seattle [30]. 

During the normal activity, i.e., without suspect of positive patients, 78% of the respondents replaced the FFP2 mask after eight hours of use, and about 62% covered the FFP2 mask with a surgical mask. This method may be considered an optimal extra precaution: as demonstrated by a recent laboratory study, the double mask method blocks 83–86% of the cough particles [31]. 

Pre-procedural mouth rinses with hydrogen peroxide and chlorhexidine, alone or used in sequence, were required by 89% of dentists. Vergara-Buenaventura et al. (2020) recommended gently gargling for 30 s in the oral cavity and 30 s in the back of the throat with 15 mL of 1.5% or 3% of hydrogen peroxide or 15 mL of 0.12% of chlorhexidine to reduce the salivary viral load [32]. The use of hydrogen peroxide and chlorhexidine in sequence appears to be a more useful measure given the conflicting results reported by some in vitro studies about the effectiveness of chlorhexidine alone for the control of COVID-19 transmission [33,34,35]. The clinical efficacy of preprocedural mouth rinse in reducing SARS-CoV-2 in dental aerosol is unclear [36]; future studies are needed. 

Changing the FFP2 mask after each aerosol-generating treatment is indicated as a more complex topic: only 22% of the dentists reported applying timely the Italian Ministry of Health directives [37]. PPE supply issue and economic costs are the more likely reasons [38,39]. New methods, such as vaporized hydrogen peroxide and ultraviolet irradiation, have been proposed to decontaminate FFP2 masks [40], although promising, the evidence of their effectiveness remains limited [41]. Furthermore, there has been encouraging evidence on the actual risk of respiratory pathogens during aerosol-generating procedures [42]. Saliva may be not considered a potential source of disease transmission during the aerosol-generating procedures, and a high-volume suction capacity air volume of 150 mm Hg or 325 L/min may be sufficient to eliminate viral contamination of the surrounding environment [42,43]. 

To further increase workplace safety, Italian dentists adopted the ventilation of the dental treatment room as a precautionary measure after each patient’s examination, regardless of the specifically performed dental treatment. About 44.86% of respondents used air sanitization devices, although these devices are expensive and non-compulsory. In rooms with poor mechanical ventilation, portable air cleaners with a high-efficiency particulate air (HEPA) filter effectively reduced aerosol accumulation and accelerated aerosol removal [11,44]. Recently, bioaerosol control devices were developed, but there is no evidence of their effectiveness in preventing airborne infections [5].

Although there exists a high level of safety and considerable trust in the current prevention procedures, Italian dentists demonstrated a great fear of possible contagion. This phenomenon was reported primarily in southern Italy, where the perception of the COVID-19 infection with all its organizational, economic, and social consequences was more dramatic than in other regions [25,45,46]. The apparent contrast among considerable confidence in precautional procedures, a low rate of contagion in dental offices, and the great reported fear may be the effect of an overwhelming sense of frustration, confusion, and anxiety [47,48] that ranges between “completely disillusioned” to “traumatized” [49].

Financial concerns were the most reported issue among Italian dentists. A recent study of the Irish Dental Association demonstrated an estimated financial loss of over 70% amid the COVID-19 outbreak [50]. In a survey carried out by the British Dental Association, 70% of dental clinics reported that they could only remain stable for three months or less [51]. Katebee et al. (2021) described that 75% of the Palestinian dentists were already facing financial hardships and could not survive financially until the end of the current month [23]. Financial loss may be strictly correlated with the reduction in the number of visits to dental offices. In our sample, more than 50% of the dentists treated less than ten patients daily. As reported by Krank et al. (2021), the reduction may have resulted from patients’ fear about contracting the virus during dental treatments and the pandemic related economic uncertainties that encouraged patients to avoid or delay dental care due to cost [52]. Longer terms of contraction may aggravate economic impact: after 135 days of upheld measures, 29% and 12% of dental practices with different levels of costs (low or high) could not cover their operative costs before taxes, thus, reporting a negative net profit over one year [53]. 

The Italian dentists expected a return to normalcy. Returning to normalcy meant more relaxation during the whole working day, reduced anxiety and fear, and a complete resumption of patient flow as before the COVID-19 outbreak. 

About a year ago, Proffitt (2020) forecasted that the ‘new’ normal would mean changes to the structure and delivery of private dentistry for some period to come. However, our study highlighted that return to normalcy has a different meaning after one year of the COVID-19 outbreak. Job insecurity and fear of COVID-19 continue to characterize the daily dental activity similar to the first days of the COVID-19 pandemic [14]. Therefore, the new normalcy results from the balance between the management of any depressive symptoms associated with uncertainty and fear [54] and the need to find solutions and compromises to adapt to the new scenario [5]. 

In this context, the complete resumption of dental activity after the COVID-19 experience has brought a new concept of safety among Italian dentists. Many precautional behaviors will be retained after the end of the COVID-19 crisis. FFP2 masks for aerosol-generating procedures, patients’ hand disinfection, ventilation of rooms, and face shields will continue to be utilized. 

From this pandemic, dentists have acquired a new perception of risk related to their everyday activity. As hypothesized by Devlin (2021), many dentists have gained a new perspective on their work [49]. Overall, dentists are now more prepared to respond to and manage potential severe and long-standing viral challenges they may face as already emerged in Nibali et al. (2020) [19]. In sudden waves due to COVID-19 variants [55], dentists are ready to assure urgent and non-urgent dental treatments with the highest level of safety. New guidelines and protocols have been recommended to reduce aerosol-generating procedures: the concept of SAFE (Safe Aerosol-Free Emergent) Dentistry [5] can be an optimal compromise to ensure dental care during the most substantial peaks of the virus waves. 

Despite significant findings, limitations have emerged. Selection bias and sampling errors are possible issues given the respondents’ non-homogenous geographical distribution, although the sample size was representative of the Italian dentists’ population. Moreover, dentists’ experience and economic characteristics were not examined (e.g., years of experience or annual income) to reduce the risk of missing values. Finally, the response rate of 41.94% was higher than the mean e-mail survey response rate of 24% or lower [56,57], but nonrespondents may differ from respondents, reducing the validity and generalizability of these results. 

## 5. Conclusions

Italy has been the first western country hit by the COVID-19 pandemic. In the last year, Italian dentists have demonstrated excellent autonomous organizational skills. Although the general uncertainty persists, Italian health professionals adopted safe and high-quality precautions to overcome the crisis and reorganize their new normalcy.

Dentists exhibited a certain level of prowess to balance the patients’ safety with operative costs. The more extended COVID-19 mitigation or suppression measures are upheld, the greater the financial distress imposed onto dental clinics will be. Governmental policies to support dental activity should be provided to aid a rapid and sustained resumption. 

This work should be used by dentists and governments worldwide to address guidelines and everyday activities to provide optimum and safe care to patients.

## Figures and Tables

**Table 1:** Respondents’ characteristics.

Characteristics	
Number of respondents	1028
Gender, *n* (%) *	
Females	289 (28.25)
Males	734 (71.75)
Age, *n* (%) **	
Less than 30 years	74 (7.21)
30–45 years	274 (26.68)
46–60 years	389 (37.88)
Over 60 years	290 (28.24)
**Geographic Area, *n* (%)**	
North-West	711 (69.16)
North-East	70 (6.81)
Center	121 (11.77)
South	95 (9.24)
Islands (Sicily and Sardinia)	31 (3.02)

* 1023/1028 interviewees reported their gender; ** 1027/1028 interviewees indicated their age range.

**Table 2:** Dental office management after the lockdown.

	Total Sample (*n* = 1028)	North-West (*n* = 711)	North-East (*n* = 70)	Center (*n* = 121)	South (*n* = 95)	Islands (*n* = 31)	*p* Value *
**A. Number of patients treated daily on average, *n* (%)**							
≤10 patients	518 (50.39)	339 (47.68)	33 (47.14)	68 (56.20)	56 (58.95)	22 (70.97)	0.092
11–20 patients	436 (42.41)	317 (44.59)	31 (44.29)	45 (37.19)	36 (37.89)	7 (22.58)	
≥20 patients	74 (7.20)	55 (7.74)	6 (8.57)	8 (6.61)	3 (3.16)	2 (6.45)	
**B. Resumption of dental activity after the lockdown, *n* (%)**							
Yes	850 (82.68)	601 (84.53)	60 (85.71)	97 (80.17)	66 (69.47)	26 (83.87)	0.006
No	178 (17.32)	110 (15.47)	10 (14.29)	24 (19.83)	29 (30.53)	5 (16.13)	

* Chi-square test.

**Table 3:** PPE and Sanitization Methods.

	Total Sample (*n* = 1028)	North-West (*n* = 711)	North-East (*n* = 70)	Center (*n* = 121)	South (*n* = 95)	Islands (*n* = 31)	*p* Value *
**A. Replacement of FFP2 mask every 8 h of use, *n* (%)**							
Yes	801 (77.92)	564 (79.32)	60 (85.71)	83 (68.60)	70 (73.68)	24 (77.42)	0.034
No	227 (22.08)	147 (20.68)	10 (14.29)	38 (31.40)	25 (26.32)	7 (22.58)	
**B. Covering of FFP2 mask with a surgical Mask, *n* (%)**							
Yes	633 (61.58)	447 (62.87)	49 (70.00)	75 (61.98)	46 (48.42)	16 (51.61)	0.028
No	395 (38.42)	264 (37.13)	21 (30.00)	46 (38.02)	49 (51.58)	15 (48.39)	
**C. Replacement of FFP2 mask after each aerosol-generating procedure, *n* (%)**							
Yes	227 (22.08)	135 (18.99)	14 (20.00)	26 (21.49)	37 (38.95)	15 (48.39)	0.000
No	801 (77.92)	576 (81.01)	56 (80.00)	95 (78.51)	58 (61.05)	16 (51.61)	
**D. Additional PPE used during aerosol-generating procedure, *n* (%)**							
Cap	876 (85.21)	612 (86.08)	61 (87.14)	105 (86.78)	75 (78.95)	23 (74.19)	0.146
Shoe covers	171 (16.63)	100 (14.06)	9 (12.86)	26 (21.49)	29 (30.53)	7 (22.58)	0.000
Double gloving	145 (14.11)	92 (12.94)	8 (11.43)	23 (19.01)	15 (15.79)	7 (22.58)	0.225
Disposable over cap	172 (16.73)	130 (18.28)	10 (14.29)	14 (11.57)	16 (16.84)	2 (6.45)	0.185
Disposable overcoat	837 (81.42)	585 (82.28)	61 (87.14)	97 (80.17)	73 (76.84)	21 (67.74)	0.129
Face Shield or protective eyewear	946 (92.02)	660 (92.83)	64 (91.43)	112 (92.56)	82 (86.32)	28 (90.32)	0.283
All PPE reported in this list	81(7.88)	50 (7.03)	4 (5.71)	8 (6.61)	13 (13.68)	6 (19.35)	0.022
**E. Room ventilation after each treatment, *n* (%)**							
Yes	915 (89.01)	624 (87.76)	64 (91.43)	108 (89.26)	91 (95.79)	28 (90.32)	0.561
No	20 (1.95)	16 (2.25)	1 (1.43)	2 (1.65)	1 (1.05)	0 (0.00)	
Only after aerosol-generating procedures	93 (9.05)	71 (9.99)	5 (7.14)	11 (9.09)	3 (3.16)	3 (9.68)	
**F. Air sanitization with special devices, *n* (%)**							
Yes	461 (44.84)	314 (44.16)	28 (40.00)	55 (45.45)	53 (55.79)	11 (35.48)	0.164
No	567 (55.16)	397 (55.84)	42 (60.00)	66 (54.55)	42 (44.21)	20 (64.52)	

* Chi-square test.

**Table 4:** Patients’ management.

	Total Sample (*n* = 1028)	North-West (*n* = 711)	North-East (*n* = 70)	Center (*n* = 121)	South (*n* = 95)	Islands (*n* = 31)	*p* Value **
**A. Patients’ management before accessing dental office** **, *n* (%)**							
Telephone triage	919 (89.40)	632 (88.89)	65 (92.86)	102 (84.30)	89 (93.68)	31 (100.00)	0.042
Hand disinfection	1023 (99.51)	709 (99.72)	69 (98.57)	121 (100.00)	93 (97.89)	31 (100.00)	0.100
Body temperature	1001 (97.37)	696 (97.89)	67 (95.71)	114 (94.21)	94 (98.95)	30 (96.77)	0.127
COVID-19 questionnaire	946 (92.02)	660 (92.83)	66 (94.29)	102 (84.30)	88 (92.63)	30 (96.77)	0.018
**B. Hand disinfection before and after accessing dental treatment room** **, *n* (%)**							
Yes	778 (76.13)	531 (75.11)	54 (77.14)	88 (73.33)	78 (82.98)	27 (87.10)	0.209
No	192 (18.79)	141 (19.94)	15 (21.43)	24 (20.00)	10 (10.64)	2 (6.45)	
Only aerosol-generating proc.	52 (5.09)	35 (4.95)	1 (1.43)	8 (6.67)	6 (6.38)	2 (6.45)	
**C. Average time of waiting in waiting room** **, *n* (%)**							
Less than 5 min	419 (40.76)	284 (39.94)	28 (40.00)	56 (46.28)	43 (45.26)	8 (25.81)	0.005
5–10 min	478 (46.50)	349 (49.09)	32 (45.71)	42 (34.71)	40 (42.11)	15 (48.39)	
10–15 min	114 (11.09)	68 (9.56)	7 (10.00)	23 (19.01)	10 (10.53)	6 (19.35)	
Over 15 min	17 (1.65)	10 (1.41)	3 (4.29)	0 (0.00)	2 (2.11)	2 (6.45)	
**D. Executed preliminary rinses** **, *n* (%)**							
Yes	914 (88.91)	637 (89.59)	60 (85.71)	105 (86.78)	86 (90.53)	26 (83.87)	0.615
**E. Types of preliminary rinses** , *n* (%) *							
Chlorhexidine (CHX)	175 (19.19)	116 (18.27)	18 (30.00)	18 (17.14)	14 (16.28)	9 (34.62)	0.003
Cetylpyridine + 0.12%CHX	96 (10.53)	55 (8.66)	5 (8.33)	21 (20.00)	12 (13.95)	3 (11.54)	
Hexetidine	0 (0.00)	0 (0.00)	0 (0.00)	0 (0.00)	0 (0.00)	0 (0.00)	
Hydrogen peroxide (H_2_O_2_)	128 (14.04)	83 (13.07)	12 (20.00)	15 (14.29)	16 (18.60)	2 (7.69)	
H_2_O_2_ and CHX	490 (53.73)	365 (57.48)	23 (38.33)	46 (43.81)	44 (51.16)	12 (46.15)	
Other	23 (2.52)	16 (2.52)	2 (3.33)	5 (4.76)	0 (0.00)	0 (0.00)	

* 912/914 interviewees responded to this question; ** Chi-square test.

**Table 5:** Contagion, positive patients, and additional precautions.

	Total Sample (*n* = 1028)	North-West (*n* = 711)	North-East (*n* = 70)	Center (*n* = 121)	South (*n* = 95)	Islands (*n* = 31)	*p* Value *
**A.COVID-19 infection**, ***n* (%)**							
Have you been infected with the SARS-CoV-2 virus?	91 (8.85)	75 (10.55)	7 (10.00)	4 (3.31)	2 (2.11)	3 (9.68)	0.013
Have any of your family members been infected?	133 (12.94)	112 (15.75)	12 (17.14)	4 (3.31)	3 (3.16)	2 (6.45)	0.000
Has any of your staff been infected?	205 (19.94)	170 (23.91)	12 (17.14)	13 (10.74)	6 (6.32)	4 (12.90)	0.000
B. Do you have employees?	860 (83.66)	598 (84.11)	59 (84.29)	100 (82.64)	75 (78.95)	28 (90.32)	0.598
Did you ask your employee to execute a serological test after the end of the lockdown?	375 (43.60)	246 (41.14)	22 (37.29)	57 (57.00)	35 (46.67)	15 (53.57)	0.025
**C.COVID-19 infection among patients**, ***n* (%)**							
Number of respondents who treated positive or suspected patients	222 (21.60)	162 (22.78)	17 (24.29)	28 (23.14)	10 (10.53)	5 (16.13)	0.075
Additional precautions to treat positive/suspected patients	64 (28.83)	44 (27.16)	7 (41.18)	6 (21.43)	4 (40.00)	3 (60.00)	0.267
**D. Number of respondents who treated patients who resulted positive after 2/3 days after the same dental treatment** **, *n* (%)**							
Yes	143 (13.91)	109 (15.33)	9 (12.86)	12 (9.92)	6 (6.32)	7 (22.58)	0.052
No	885 (86.09)	602 (84.67)	61 (87.14)	109 (90.08)	89 (93.68)	24 (77.42)	
**E. Number of respondents who contact the competent doctor after treated patients resulted positive after 2/3 days after the dental treatment** **, *n* (%)**	74 (51.75)	54 (49.54)	6 (66.67)	6 (50.00)	5 (83.33)	3 (42.86)	0.456
**Additional precautions suggested by the competent doctor**							
Only rapid test	10 (13.51)	5 (9.43)	3 (25.00)	1 (25.00)	0 (0.00)	1 (25.00)	0.053
Fiduciary isolation	1 (1.35)	1 (1.89)	0 (0.00)	0 (0.00)	0 (0.00)	0 (0.00)	0.985
Rapid test and fiduciary isolation	15 (20.27)	7 (13.21)	1 (8.33)	2 (50.00)	5 (100.00)	0 (0.00)	0.000
None	46 (62.16)	40 (75.47)	2 (16.67)	2 (50.00)	0 (0.00)	2 (50.00)	0.004
Other	2 (2.70)	2 (3.77)	0 (0.00)	0 (0.00)	0 (0.00)	0 (0.00)	NA
**Additional precautions autonomously adopted**							
Only rapid test	16 (23.19)	16 (29.09)	0 (0.00)	0 (0.00)	0 (0.00)	0 (0.00)	0.000
Fiduciary isolation	1 (1.45)	1 (1.82)	0 (0.00)	0 (0.00)	0 (0.00)	0 (0.00)	0.406
Rapid test and fiduciary isolation	5 (7.25)	4 (7.27)	0 (0.00)	1 (16.67)	0 (0.00)	0 (0.00)	0.017
None	45 (65.22)	32 (58.18)	3 (100.00)	5 (83.33)	1 (100.00)	4 (100.00)	0.000
Other	2 (2.90)	2 (3.64)	0 (0.00)	0 (0.00)	0 (0.00)	0 (0.00)	0.092

* Chi-square test.

**Table 6:** Perceptions scores expressed as mean (standard deviation)—total sample and geographic area.

	Total Sample (*n* = 1028)	North-West (*n* = 711)	North-East (*n* = 70)	Centre (*n* = 121)	South (*n* = 95)	Islands (*n* = 31)	*p* Value *
I am worried that I may contract the virus during my work	3.06 (1.33)	2.98 (1.32)	2.83 (1.43)	3.17 (1.25)	3.55 (1.40)	3.45 (1.18)	0.0003
Given the current pandemic situation, I would like to suspend my business	1.58 (1.07)	1.52 (1.02)	1.47 (0.94)	1.57 (1.04)	2.03 (1.36)	1.77 (1.15)	0.0013
I am worried about being in quarantine or fiduciary isolation	3.74 (1.36)	3.71 (1.36)	3.64 (1.60)	3.74 (1.26)	3.99 (1.29)	3.84 (1.37)	0.3158
I believe that in 2021 I will continue to have economic losses	3.54 (1.32)	3.44 (1.32)	3.31 (1.37)	3.66 (1.34)	4.17 (1.08)	3.96 (1.11)	0.0001
I am confident in current contagion prevention procedures	3.90 (1.10)	3.93 (1.10)	4.07 (1.01)	3.76 (1.14)	3.73 (1.21)	3.68 (0.94)	0.0713

* Kruskal Wallis test with Dunn’s procedure was used for comparisons.

**Table 7:** Perceptions scores expressed as mean (standard deviation) per age range.

	Less Than 30 Years	30–45 Years	46–60 Years	More Than 60 Years	*p* Value *
I am worried that I may contract the virus during my work	2.97 (1.15)	2.87 (1.35)	3.05 (1.33)	3.26 (1.33)	0.0076
Given the current pandemic situation, I would like to suspend my business	1.45 (0.81)	1.53 (1.02)	1.51 (1.04)	1.74 (1.17)	0.0066
I am worried about being in quarantine or fiduciary isolation	3.54 (1.28)	3.74 (1.35)	3.83 (1.36)	3.66 (1.39)	0.1319
I believe that in 2021 I will continue to have economic losses	3.12 (1.17)	3.07 (1.36)	3.65 (1.27)	3.94 (1.20)	0.0001
I am confident in current contagion prevention procedures	3.74 (1.09)	3.78 (1.17)	4.05 (1.03)	3.83 (1.12)	0.0038

* Kruskal Wallis test with Dunn’s procedure was used for comparisons.

**Table 8:** Return to normalcy.

	Total Sample (*n* = 1028)	North-West (*n* = 711)	North-East (*n* = 70)	Center (*n* = 121)	South (*n* = 95)	Islands (*n* = 31)	*p* Value *
**A. Do you believe that we return to normalcy as that before the** **COVID-19 outbreak? *n* (%)**							
Yes	505 (49.12)	334 (46.98)	35 (50.00)	64 (52.89)	54 (56.84)	18 (58.06)	0.260
No	523 (50.88)	377 (53.02)	35 (50.00)	57 (47.11)	41 (43.16)	13 (41.94)	
**B. When the** **COVID-19 pandemic is over or under control, will you adopt some or all of the preventive behaviors you learned during this pandemic? (Total sample: *n* = 505) *n* (%)**							
Yes	459 (90.89)	300 (89.82)	33 (94.29)	59 (92.19)	50 (92.59)	17 (94.44)	0.819
No	46 (9.11)	34 (10.18)	2 (5.71)	5 (7.81)	4 (7.41)	1 (5.56)	
**C. What behaviors you learned during the pandemic will you continue to adopt? *n* (%)**							
Patients hand disinfection	349 (33.95)	225 (31.65)	25 (35.71)	50 (41.32)	35 (36.84)	14 (45.16)	0.405
FFP2 mask (even just for aerosol-generating procedures)	337 (32.78)	207 (29.11)	22 (31.43)	53 (43.80)	38 (40.00)	17 (54.84)	0.001
COVID-19 questionnaire	91 (8.85)	57 (8.02)	4 (5.71)	18 (14.88)	9 (9.47)	3 (9.68)	0.218
Air sanitization with specific devices	191 (18.58)	123 (17.30)	15 (21.43)	26 (21.49)	20 (21.05)	7 (22.58)	0.978
Preliminary rinses with hydrogen peroxide	226 (21.98)	145 (20.39)	13 (18.57)	32 (26.45)	25 (26.32)	11 (35.48)	0.462
Disposable overcoat	192 (18.68)	127 (17.86)	10 (14.29)	27 (22.31)	21 (22.11)	7 (22.58)	0.697
Thermo scanner	158 (15.37)	103 (14.49)	6 (8.57)	25 (20.66)	15 (15.79)	9 (29.03)	0.074
Telephone triage	156 (15.18)	105 (14.77)	10 (14.29)	20 (16.53)	14 (14.74)	7 (22.58)	0.822
Room ventilation	390 (37.94)	252 (35.44)	32 (45.71)	52 (42.98)	42 (44.21)	12 (38.71)	0.126
Face shield	377 (36.67)	240 (33.76)	29 (41.43)	54 (44.63)	43 (45.26)	11 (35.48)	0.055
**D. What does it mean for you to go back to normal?** ***n* (%)**							
Greater relaxation during the entire daily activity	334 (32.49)	219 (30.80)	25 (35.71)	44 (36.36)	35 (36.84)	11 (35.48)	0.940
Reduction in the level of anxiety/fear of being infected	257 (25.00)	153 (21.52)	17 (24.29)	41 (33.88)	34 (35.79)	12 (38.71)	0.008
Restoration of patient flow as in the pre-COVID period	294 (28.60)	190 (26.72)	22 (31.43)	39 (32.23)	34 (35.79)	9 (29.03)	0.847
Use of noninvasive sanitation procedures	148 (14.40)	99 (13.92)	10 (14.29)	24 (19.83)	11 (11.58)	4 (12.90)	0.317

* Chi-square test.

## Data Availability

Not applicable.

## Supplementary Materials

The following are available online at https://www.mdpi.com/article/10.3390/dj9080086/s1, Table S1: Questionnaire [5,11,21,22,25,26,28,29,30,31,32,33,34,35,37,41,55]; Table S2: Number of dentists by geographic areas.

## References

[1] World Health Organization Italy Situation. https://covid19.who.int/region/euro/country/it.

[2] Scully C., Samaranayake L.P. (2016). Emerging and Changing Viral Diseases in the New Millennium. Oral Dis..

[3] Harrel S.K., Molinari J. (2004). Aerosols and Splatter in Dentistry: A Brief Review of the Literature and Infection Control Implications. J. Am. Dent. Assoc..

[4] Occupational Safety and Health Administration Covid-19 Control and Prevention—Dentistry Workers and Employers. https://www.osha.gov/coronavirus/control-prevention/dentistry.

[5] Beltrán-Aguilar E., Benzian H., Niederman R. (2021). Rational Perspectives on Risk and Certainty for Dentistry during the COVID-19 Pandemic. Am. J. Infect. Control..

[6] Centers for Disease Control and Prevention (CDC) Interim Infection Prevention and Control Guidance for Dental Settings during the Coronavirus Disease 2019 (COVID-19) Pandemic. https://www.cdc.gov/coronavirus/2019-ncov/hcp/dental-settings.html.

[7] American Dental Association (ADA) Return to Work Interim Guidance Toolkit. https://pages.ada.org/return-to-work-toolkit-american-dental-association.

[8] Silva W.O., Vianna Silva Macedo R.P., Nevares G., Val Rodrigues R.C., Grossi Heleno J.F., Braga Pintor A.V., Almeida B.M. (2021). Recommendations for Managing Endodontic Emergencies during Covid-19 Outbreak. J. Endod..

[9] Barabari P., Moharamzadeh K. (2020). Novel Coronavirus (Covid-19) and Dentistry–a Comprehensive Review of Literature. Dent. J..

[10] Ren Y., Feng C., Rasubala L., Malmstrom H., Eliav E. (2020). Risk for Dental Healthcare Professionals during the COVID-19 Global Pandemic: An Evidence-Based Assessment. J. Dent..

[11] Ren Y.F., Rasubala L., Malmstrom H., Eliav E. (2020). Dental Care and Oral Health under the Clouds of COVID-19. JDR Clin. Transl. Res..

[12] Woloshin S., Patel N., Kesselheim A.S. (2020). False Negative Tests for SARS-CoV-2 Infection—Challenges and Implications. N. Engl. J. Med..

[13] Governo Italiano Coronavirus, Le Misure Adottate Dal Governo. http://www.governo.it/it/coronavirus-misure-del-governo.

[14] Salgarello S., Salvadori M., Mazzoleni F., Salvalai V., Francinelli J., Bertoletti P., Lorenzi D., Audino E., Garo M.L. (2020). Urgent Dental Care During Italian Lockdown: A Cross-Sectional Survey. J. Endod..

[15] De Stefani A., Bruno G., Mutinelli S., Gracco A. (2020). COVID-19 Outbreak Perception in Italian Dentists. Int. J. Environ. Res. Public Health.

[16] Ministero della Salute Indicazioni Operative per L’attività Odontoiatrica Durante La Fase 2 Della Pandemia Covid-19. http://www.salute.gov.it/imgs/C_17_pubblicazioni_2917_allegato.pdf.

[17] Ordine dei Medici di Brescia The Cassandra Crossing. https://www.ordinemedici.brescia.it/archivio10_notizie-e-comunicati_1_2240.html.

[18] Bakaeen L.G., Masri R., AlTarawneh S., Garcia L.T., AlHadidi A., Khamis A.H., Hamdan A.M., Baqain Z.H. (2021). Dentists’ Knowledge, Attitudes, and Professional Behavior toward the COVID-19 Pandemic: A Multisite Survey of Dentists’ Perspectives. J. Am. Dent. Assoc..

[19] Nibali L., Ide M., Ng D., Buontempo Z., Clayton Y., Asimakopoulou K. (2020). The Perceived Impact of Covid-19 on Periodontal Practice in the United Kingdom: A Questionnaire Study. J. Dent..

[20] ISTAT Descrizione Dei Dati Geografici Dei Confini Delle Unità Amministrative a Fini Statistici. https://www.istat.it/it/files//2013/11/2019.28.06-Descrizione-dei-dati.pdf.

[21] Bellini P., Checchi V., Liani C., Bencivenni D., Consolo U. (2020). Psychological Reactions to COVID-19 and Epidemiological Aspects of Dental Practitioners during Lockdown in Italy. Minerva Stomatol..

[22] Consolo U., Bellini P., Bencivenni D., Iani C., Checchi V. (2020). Epidemiological Aspects and Psychological Reactions to COVID-19 of Dental Practitioners in the Northern Italy Districts of Modena and Reggio Emilia. Int. J. Environ. Res. Public Health.

[23] Kateeb E.T., Juniedi R.N., Warren J.J. (2021). Reopening Dental Offices for Routine Care Amid the COVID-19 Pandemic: Report From Palestine. Int. Dent. J..

[24] American Dental Association One Year of COVID-19 Response. https://www.ada.org/en/publications/ada-news/2021-archive/march/one-year-of-covid19-response.

[25] Gatto M., Bertuzzo E., Mari L., Miccoli S., Carraro L., Casagrandi R., Rinaldo A. (2020). Spread and Dynamics of the COVID-19 Epidemic in Italy: Effects of Emergency Containment Measures. Proc. Natl. Acad. Sci. USA..

[26] Estrich C.G., Mikkelsen M., Morrisey R., Geisinger M.L., Ioannidou E., Vujicic M., Araujo M.W.B. (2020). Estimating COVID-19 Prevalence and Infection Control Practices among US Dentists. J. Am. Dent. Assoc..

[27] Putrino A., Raso M., Magazzino C., Galluccio G. (2020). Coronavirus (COVID-19) in Italy: Knowledge, Management of Patients and Clinical Experience of Italian Dentists during the Spread of Contagion. BMC Oral Health.

[28] Kluytmans-van den Bergh M.F.Q., Buiting A.G.M., Pas S.D., Bentvelsen R.G., van den Bijllaardt W., van Oudheusden A.J.G., van Rijen M.M.L., Verweij J.J., Koopmans M.P.G., Kluytmans J.A.J.W. (2020). Prevalence and Clinical Presentation of Health Care Workers With Symptoms of Coronavirus Disease 2019 in 2 Dutch Hospitals During an Early Phase of the Pandemic. JAMA Netw. Open.

[29] Lai X., Wang M., Qin C., Tan L., Ran L., Chen D., Zhang H., Shang K., Xia C., Wang S. (2020). Coronavirus Disease 2019 (COVID-2019) Infection Among Health Care Workers and Implications for Prevention Measures in a Tertiary Hospital in Wuhan, China. JAMA Netw. Open.

[30] Mani N.S., Budak J.Z., Lan K.F., Bryson-Cahn C., Zelikoff A., Barker G.E.C., Grant C.W., Hart K., Barbee C.J., Sandoval M.D. (2020). Prevalence of COVID-19 Infection and Outcomes Among Symptomatic Healthcare Workers in Seattle, Washington. Clin. Infect. Dis. Off. Publ. Infect. Dis. Soc. Am..

[31] Brooks J.T., Beezhold D.H., Noti J.D., Coyle J.P., Derk R.C., Blachere F.M., Lindsley W.G. (2021). Maximizing Fit for Cloth and Medical Procedure Masks to Improve Performance and Reduce SARS-CoV-2 Transmission and Exposure, 2021. MMWR Morb. Mortal. Wkly. Rep..

[32] Vergara-Buenaventura A., Castro-Ruiz C. (2020). Use of Mouthwashes against COVID-19 in Dentistry. Br. J. Oral Maxillofac. Surg..

[33] Peng X., Xu X., Li Y., Cheng L., Zhou X., Ren B. (2020). Transmission Routes of 2019-NCoV and Controls in Dental Practice. Int. J. Oral Sci..

[34] Kampf G., Todt D., Pfaender S., Steinmann E. (2020). Persistence of Coronaviruses on Inanimate Surfaces and Their Inactivation with Biocidal Agents. J. Hosp. Infect..

[35] Yoon J.G., Yoon J., Song J.Y., Yoon S.Y., Lim C.S., Seong H., Noh J.Y., Cheong H.J., Kim W.J. (2020). Clinical Significance of a High SARS-CoV-2 Viral Load in the Saliva. J. Korean Med. Sci..

[36] Reis I.N.R., do Amaral G.C.L.S., Mendoza A.A.H., das Graças Y.T., Mendes-Correa M.C., Romito G.A., Pannuti C.M. (2021). Can Preprocedural Mouthrinses Reduce SARS-CoV-2 Load in Dental Aerosols?. Med. Hypotheses.

[37] American Dental Association (ADA) HPI Poll: Dentists See ‘Substantial’ Increase in PPE Prices. https://www.ada.org/en/publications/ada-news/2020-archive/december/hpi-poll-dentists-see-substantial-increase-in-ppe-prices.

[38] Rossato M.D.S., Gregorio D., de Almeida-Pedrin R.R., Maia L.P., Poli R.C., Berger S.B., Fernandes T.M.F. (2021). Evaluation of Dental Practices Changes During the COVID-19 Pandemic in Brazil. Eval. Health Prof..

[39] Cavalcanti Y.W., da Silva R.O., de Ferreira L.F., de Lucena E.H.G., de Souza A.M.L.B., de Cavalcante D.F.B., de Meneghim M.C., Pereira A.C. (2020). Economic Impact of New Biosafety Recommendations for Dental Clinical Practice during Covid-19 Pandemic. Pesqui. Bras. Odontopediatria Clin. Integr..

[40] Ludwig-Begall L.F., Wielick C., Dams L., Nauwynck H., Demeuldre P.F., Napp A., Laperre J., Haubruge E., Thiry E. (2020). The Use of Germicidal Ultraviolet Light, Vaporized Hydrogen Peroxide and Dry Heat to Decontaminate Face Masks and Filtering Respirators Contaminated with a SARS-CoV-2 Surrogate Virus. J. Hosp. Infect..

[41] Sarkis-Onofre R., do Borges R.C., Demarco G., Dotto L., Schwendicke F., Demarco F.F. (2021). Decontamination of N95 Respirators against SARS-CoV-2: A Scoping Review. J. Dent..

[42] Meethil A.P., Saraswat S., Chaudhary P.P., Dabdoub S.M., Kumar P.S. (2021). Sources of SARS-CoV-2 and Other Microorganisms in Dental Aerosols. J. Dent. Res..

[43] Akin H., Karabay O., Toptan H., Furuncuoglu H., Kaya G., Akin E.G., Koroglu M. (2021). Investigation of the Presence of SARS-CoV-2 in Aerosol after Dental Treatment. Int. Dent. J..

[44] Ren Y.F., Huang Q., Marzouk T., Richard R., Pembroke K., Martone P., Venner T., Malmstrom H., Eliav E. (2021). Effects of Mechanical Ventilation and Portable Air Cleaner on Aerosol Removal from Dental Treatment Rooms. J. Dent..

[45] Ferre F., de Belvis A.G., Valerio L., Longhi S., Lazzari A., Fattore G., Ricciardi W., Maresso A. (2014). Italy: Health System Review. Health Syst. Transit..

[46] Rossi R., Socci V., Talevi D., Mensi S., Niolu C., Pacitti F., di Marco A., Rossi A., Siracusano A., di Lorenzo G. (2020). COVID-19 Pandemic and Lockdown Measures Impact on Mental Health Among the General Population in Italy. Front. Psychiatry.

[47] Mijiritsky E., Hamama-Raz Y., Liu F., Datarkar A.N., Mangani L., Caplan J., Shacham A., Kolerman R., Mijiritsky O., Ben-Ezra M. (2020). Subjective Overload and Psychological Distress among Dentists during COVID-19. Int. J. Environ. Res. Public Health.

[48] Tysiąc-Miśta M., Dziedzic A. (2020). The Attitudes and Professional Approaches of Dental Practitioners during the COVID-19 Outbreak in Poland: A Cross-Sectional Survey. Int. J. Environ. Res. Public Health.

[49] Devlin A.C. (2021). Post-Pandemic Dentistry—Restart or Reform?. Br. Dent. J..

[50] Irish Dental Association Irish Dental Association Warns the Profession is on the Brink of Collapse. https://www.dentistrytoday.com/news/industrynews/item/6156-irish-dental-association-warns-the-profession-is-on-the-brink-of-collapse.

[51] British Dental Association Practices Weeks from Collapse without Rapid Action from Government. https://www.bda.org/news-centre/press-releases/Pages/Practices-months-from-collapse-without-rapid-action-from-UK-government.aspx.

[52] Kranz A.M., Chen A., Gahlon G., Stein B.D. (2021). 2020 Trends in Dental Office Visits during the COVID-19 Pandemic. J. Am. Dent. Assoc..

[53] Schwendicke F., Krois J., Gomez J. (2020). Impact of SARS-CoV2 (Covid-19) on Dental Practices: Economic Analysis. J. Dent..

[54] Gasparro R., Scandurra C., Maldonato N.M., Dolce P., Bochicchio V., Valletta A., Sammartino G., Sammartino P., Mariniello M., di Lauro A.E. (2020). Perceived Job Insecurity and Depressive Symptoms among Italian Dentists: The Moderating Role of Fear of Covid-19. Int. J. Environ. Res. Public Health.

[55] Arif T. (2021). bin The 501.V2 and B.1.1.7 Variants of Coronavirus Disease 2019 (COVID-19): A New Time-Bomb in the Making?. Infect. Control. Hosp. Epidemiol..

[56] Shih T.H., Fan X. (2009). Comparing Response Rates in E-Mail and Paper Surveys: A Meta-Analysis. Educ. Res. Rev..

[57] Sheehan K.B. (2006). E-Mail Survey Response Rates: A Review. J. Comput. Mediat. Commun..

