# Supplementary material for: The New Normalcy in Dentistry after the COVID-19 Pandemic: An Italian Cross-Sectional Survey
Source: Dent J (Basel). 2021 Jul 31;9(8):86. doi: 10.3390/dj9080086 (PMC8391335; doi:10.3390/dj9080086)
Supplement: Supplementary file 1 [file dentistry-09-00086-s001.zip › dentistry-1277015-supplementary.pdf]

Table S1. Questionnaire.

Num.	Question	Answers
**1**	How many patients do you visit daily on average?	• ≤ 10 patients<br>• 11 – 20 patients<br>• ≥ 20 patients
**2**	From the end of the Italian lockdown, have you restarted your regular activity?	• Yes<br>• No
**3**	Do you replace the FFP2 mask after every 8 hours of use, even if not continuously?	• Yes<br>• No
**4**	Do you simultaneously wear an FFP2 mask and surgical mask?	• Yes<br>• No
**5**	Do you replace the FFP2 mask after each aerosol-generating procedure?	• Yes<br>• No
**6**	What PPE do you wear during aerosol-generating procedures?*	• Head covering<br>• Shoe covers<br>• Double gloving<br>• All PPE reported in this list<br>• Others
**7**	Do you ventilate dental treatment rooms after each dental treatment?	• Yes<br>• No<br>• Only after aerosol-generating procedures
**8**	After each aerosol-generating procedure, do you sanitize air with special devices?	• Yes<br>• No
**9**	Do you execute telephone triage for each patient?	• Yes<br>• No
**10**	Do you ask patients to clean their hands before accessing the dental office?	• Yes<br>• No
**11**	Do you measure patients' body temperature before accessing the dental office?	• Yes<br>• No
**12**	Do you ask patients to complete the prescreening Covid-19 questionnaire?	• Yes<br>• No
**13**	Do you ask patients to clean their hands again before accessing the dental treatment room?	• Yes<br>• No
**14**	What is, on average, the time of permanence in the waiting room for each patient?	• Less than 5 minutes<br>• 5-10 minutes<br>• 10-15 minutes<br>• Over 15 minutes
**15**	Before dental treatment, do you ask the patient to rinse their mouth?	• Yes<br>• No
**16**	What kind of rinses do you do before dental treatments?	• Chlorhexidine (CHX)<br>• Cetylpyridine(CPC) with 0.12% CHX<br>• Esetidine<br>• Hydrogen peroxide (H_2O_2)<br>• H_2O_2 followed by Chlorhexidine<br>• Other
**17**	Have you been infected with the SARS-CoV-2 virus?	• Yes<br>• No
**18**	Have any of your family members been infected?	• Yes<br>• No
**19**	Has any of your staff been infected?	• Yes

		• No
**20**	Have you treated Covid-19 positive or suspected patients?	• Yes<br>• No
**21**	Did you adopt other PPEs, sanitization, or ventilation methods treating Covid-19 positive/suspected patients?	• Yes<br>• No
**22**	Do you have employees?	• Yes<br>• No
**23**	Did you ask your employees to perform a serological test after the end of the lockdown?	• Yes<br>• No
**24**	Did you treat patients who resulted Covid-19 positive after one/two days after dental treatment?	• Yes<br>• No
**25**	After the patient's positive communication, did you contact the competent doctor to decide if and what precautions to take?	• Yes<br>• No
**26**	What precautions did you or the competent doctor adopt?	• Only rapid test<br>• Only fiduciary isolation for staff who came in contact with the infected patient<br>• Rapid test and fiduciary isolation<br>• No precautions because the infection could not have occurred due to procedures and precautions taken during the dental treatment<br>• Other
**27**	I am worried that I may contract the virus during my work	• 1 = Strongly disagree<br>• 2 = Disagree<br>• 3 = Neither agree or disagree<br>• 4 = Agree<br>• 5 = Strongly agree
**28**	Given the current pandemic situation, I would like to suspend my business	• 1 = Strongly disagree<br>• 2 = Disagree<br>• 3 = Neither agree nor disagree<br>• 4 = Agree<br>• 5 = Strongly agree
**29**	I am worried about being in quarantine or fiduciary isolation	• 1 = Strongly disagree<br>• 2 = Disagree<br>• 3 = Neither agree nor disagree<br>• 4 = Agree<br>• 5 = Strongly agree
**30**	I believe that in 2021 I will continue to have economic losses	• 1 = Strongly disagree<br>• 2 = Disagree<br>• 3 = Neither agree nor disagree<br>• 4 = Agree<br>• 5 = Strongly agree
**31**	I am confident in current contagion prevention procedures	• 1 = Strongly disagree<br>• 2 = Disagree<br>• 3 = Neither agree nor disagree<br>• 4 = Agree<br>• 5 = Strongly agree
**32**	Do you think that we will return to normalcy as that before the Covid-19 outbreak?	• Yes<br>• No
**33**	When the Covid-19 pandemic is over or under control, will you adopt some or all of the preventive behaviors you learned during this pandemic?	• Yes<br>• No

**34**	What behaviors you learned during the pandemic will you continue to adopt?*	• Patients hand disinfection<br>• FFP2 mask (even just for aerosol-generating procedures)<br>• Covid-19 questionnaire<br>• Air sanitization with specific devices<br>• Preliminary rinses with hydrogen peroxide<br>• Disposable overcoat<br>• Thermo scanner<br>• Telephone triage<br>• Air ventilation<br>• Face shield
**35**	What does it mean for you to go back to normal?	• Greater relaxation during the entire daily activity<br>• Reduction in the level of anxiety/fear of being infected<br>• Restoration of patient flow as in the pre-Covid period<br>• Use of noninvasive sanitation procedures
**36**	Gender	• Female<br>• Male
**37**	Age	• Less than 30 years<br>• 30 – 45<br>• 46 – 60<br>• Over 60 years
**38**	Region	Free-text

* Multiple-choice response

Table S2. Number of dentists by geographic areas.

**Number of dentists by geographic areas.**	
**Total Number**	**58,579**
**Geographic Area, n(%)**	
North-West	16,634
North-East	11,196
Center	13,151
South	12,076
Islands (Sicily and Sardinia)	5,522

* Source Information: Putrino et al. (2020)
